# Access to clinical data for direct oral anticoagulant prescriptions in community pharmacy and implications for medication optimisation: a mixed-methods study

**DOI:** 10.1007/s11096-026-02159-3

**Published:** 2026-05-22

**Authors:** Jacqueline G. van Paassen, Vera H. M. Deneer, Thomas G. H. Kempen, Marcel L. Bouvy

**Affiliations:** 1 Division of Pharmacoepidemiology and Clinical Pharmacology, Utrecht Institute for Pharmaceutical Sciences, Utrecht University, Utrecht, The Netherlands; 2 Department of Clinical Pharmacy, Division Laboratories, Pharmacy and Biomedical Genetics, University Medical Centre Utrecht, Utrecht, The Netherlands; 3 Department of Pharmacy, Uppsala University, Uppsala, Sweden

**Keywords:** Anticoagulants, Clinical reasoning, Community pharmacists, Inappropriate prescribing, Medication errors, Patient care

## Abstract

**Introduction:**

Inappropriate direct oral anticoagulant (DOAC) prescribing is a frequent cause of harm to patients. Pharmacists could play an important role in optimising prescribing, but they need access to relevant clinical data to do so. Little is known about community pharmacists’ access to clinical data to assess the appropriateness of DOAC prescriptions.

**Aim:**

To investigate the availability of relevant clinical data for DOAC prescriptions and whether additionally requesting such data supports community pharmacists in assessing and optimising DOAC prescriptions. This study also explored behavioural factors influencing pharmacists’ decisions to request additional clinical data.

**Method:**

In this mixed-methods study, 166 community pharmacies were approached to collect quantitative data. This included the prevalence of missing clinical data, pharmacists’ requests for missing data including reasons for requesting these data, and prescription adjustments made by the pharmacist. Missing clinical data was defined as absence of patient information on the DOAC prescription to assess its appropriateness: treatment indication, renal function, body weight, treatment duration, or concomitant use of antithrombotics. A random sample of 10 participating pharmacies was approached for semi-structured interviews on perceptions whether to request missing clinical data. Quantitative data were analysed descriptively. Interviews were thematically analysed using the Capability, Opportunity, Motivation, Behaviour (COM-B) framework to understand behaviour. Quantitative and qualitative data were triangulated through discussion of the findings.

**Results:**

We collected quantitative data of 583 DOAC prescriptions in 92 pharmacies and interviewed 11 pharmacists from 10 approached pharmacies. On 92.8% (n = 541) of DOAC prescriptions clinical data were missing. Additional clinical data (particularly, treatment indication and renal function) were requested in 51.9% (n = 281), mainly for dose evaluation (39.6% (n = 231)), leading to 5.0% (n = 14) adjustments of these prescriptions. Pharmacists applied clinical reasoning to determine the need for additional data, with providing optimal patient care as their primary motivation. The main opportunity was collaboration with other healthcare professionals.

**Conclusion:**

Using their clinical reasoning competence, pharmacists frequently requested missing clinical data, particularly treatment indication and renal function, to verify DOAC prescriptions and to ensure optimal patient care. Clinical data sharing could be optimised by streamlined collaboration between healthcare professionals.

**Supplementary Information:**

The online version contains supplementary material available at https://doi.org/10.1007/s11096-026-02159-3.

## Impact statements

- Our findings imply that direct oral anticoagulant (DOAC) prescriptions should consistently include treatment indication and renal function to assess its appropriateness.
- To determine whether missing clinical data should be requested and to ensure optimal pharmaceutical care, pharmacists should apply clinical reasoning skills.
- Streamlined communication between healthcare professionals, including community pharmacists, supports agreements on sharing clinical data to optimise safe and effective use of DOACs.

## Introduction

Direct oral anticoagulants (DOACs) have rapidly become the first-choice treatment for the prevention of thrombosis in multiple clinical conditions, such as atrial fibrillation (AF), and deep vein thrombosis and pulmonary embolism [1–3]. Despite their advantages in terms of efficacy and safety, appropriate DOAC prescribing for individual patients is challenging [4]. Various dosages exist for a given indication, and several patient-related dose reduction criteria (e.g. age, renal function and body weight) along with potential drug-drug interactions require a careful approach by healthcare professionals [4–6].

Patient-related factors and other relevant clinical data are frequently insufficiently considered when prescribing DOACs [5]. Studies using real-world data report that the prevalence of inappropriate DOAC dosing in AF patients varies between 6 and 50% [7–10]. Underdosing may increase the risk of all-cause mortality, whereas overdosing is associated with an increased risk of major bleeding [6]. As a result, awareness among all healthcare professionals involved in the prescribing and dispensing process is essential to identify and minimise potentially inappropriate dosing. In this context, community pharmacists in the Netherlands are responsible for both provision of pharmaceutical care and dispensing medications in primary care [11]. During the dispensing process, pharmacists routinely assess the appropriateness of DOAC prescriptions (estimated to involve 620,000 users in 2024 [12]) and dosing based on clinical data such as renal function, treatment indication, and concomitant medication use. Sometimes, these clinical data may be included directly on the prescription or be available in the pharmacy’s information system. In other cases, pharmacists need to consult the prescriber or the patient to obtain the necessary information. If the dosing or overall prescription is considered inappropriate, the pharmacist consults the prescriber to discuss the appropriateness and, if applicable, to adjust the prescription.

Given the potential harm associated with inappropriate DOAC dosing, measures are needed to reduce these errors. Commonly suggested strategies are pharmacist interventions or multidisciplinary stewardship programs [5, 13–15]. For such interventions or programs to be effective, all involved healthcare professionals (HCPs) should have access to relevant clinical data, such as the indication for DOAC therapy and renal function, as this may reduce inappropriate dosing of antithrombotic treatment [16–18]. Such access should be regarded as a valuable component of multidisciplinary collaboration [19]. From HCP perspectives, multidisciplinary collaboration, fewer operational challenges, such as high workload and too little time, and reliable information can reduce inappropriate DOAC dosing [19–21].

To date, little is known about Dutch community pharmacists’ access to clinical data when processing DOAC prescriptions in primary care, and the extent to which the availability of such clinical data leads to adjustments in those prescriptions. Furthermore, we are unaware of the pharmacists’ perceptions, barriers and facilitators on the availability and use of clinical data.

## Aim

This study aimed to investigate the availability of relevant clinical data for DOAC prescriptions in primary care and whether additionally requesting clinical data supports community pharmacists in assessing and optimising DOAC prescriptions. This study also explored behavioural factors influencing pharmacists’ decisions to request additional clinical data.

## Method

### Study design and setting

This study employed a mixed-methods embedded design, integrating quantitative data on the assessment of DOAC prescriptions in community pharmacies in the Netherlands with qualitative data based on interviews with a selected sample of pharmacists [22]. This approach enhanced insight into the underlying reasoning for requesting clinical data and the barriers and facilitators influencing pharmacists’ decision-making regarding such requests. We designed the aim and parts of the methodology based on input from practicing community pharmacists, who identified key challenges in processing DOAC prescriptions in current pharmacy practice.

We applied the Mixed Methods Appraisal Tool (MMAT) and the Consolidated criteria for reporting qualitative research (COREQ; Online Resource 1) to guide study design and reporting [23, 24].

### Quantitative data of DOAC prescriptions

#### Participants

Community pharmacists were approached through the Utrecht Pharmacy Practice network for Education and Research (UPPER) [25]. The UPPER network is a network of Dutch community and hospital pharmacies with an interest in research and education. An email was sent to community pharmacies in the UPPER network in close proximity of Utrecht (n = 88) with a request to participate in our study. Additionally, all community pharmacies expected to host a Master of Pharmacy student for their internship in the community pharmacy (also coincidentally n = 88; all part of the UPPER network) were contacted. Ten of these pharmacies belonged to both groups, resulting in a total of 166 unique pharmacies invited.

#### Eligible prescriptions

We included first-time DOAC prescriptions (apixaban, dabigatran, edoxaban or rivaroxaban) of individual patients. A first-time prescription was defined as the first dispensing of a specific DOAC to a specific patient at the participating pharmacy, irrespective of previous dispensing at hospital discharge, the outpatient hospital pharmacy (e.g. after an outpatient visit to the hospital), or another community pharmacy. No prescriptions were excluded.

#### Outcome measures

The main outcomes were the prevalence of missing clinical data included on first-time DOAC prescriptions, pharmacists’ requests for additional data, and prescription adjustments by pharmacists after consultation with the prescriber to optimise DOAC therapy. Missing clinical data was defined based on the dose defining criteria from the EHRA guide on DOACs and input from practicing community pharmacists in the Netherlands [4]. Its definition was absence of at least one of the following parameters to assess appropriateness of DOAC prescriptions: treatment indication, renal function, body weight, treatment duration, or concomitant use of antithrombotics. Secondary outcomes included differences in missing data and data requests by prescriber type (general practitioner (GP) vs. medical specialist), as well as the source of additional information the pharmacist used to request additional data (prescriber, patient, or pharmacy information system) and the reasons for requesting these additional data. From our prior research we identified these secondary outcomes as relevant [18].

#### Data collection

Data were collected using a collection form (Online Resource 2) by participating pharmacists or Master of Pharmacy students during their internship in the participating pharmacies between February and September 2023. This collection form was based on input from practicing community pharmacists, and was pilot tested by Master of Pharmacy students during their internship (in January 2023). The form contained questions about the DOAC prescription: general data, patient data, type of prescriber, clinical data included on the prescription and clinical data requested, and specific questions about assessing the prescription and drug dispensing, including underlying reasoning to request clinical data. The clinical data included treatment indication, renal function, body weight, duration of use, and concomitant use of antithrombotics.

The collection form was available both online (using Qualtrics XM) and offline (pdf format). The participant could choose which way of collection was preferred. If the participant chose the offline version, the completed forms were sent to the researchers monthly via a secured data platform and imported into Qualtrics XM. All data was ultimately collected in Qualtrics XM as the primary data source.

#### Data analysis

We verified data reliability by checking each data entry for completeness and inconsistencies (including free-text fields if informative) and requested additional prescription details from participants, if needed. Duplicates, identified through pharmacy identifier, date of birth, and registration date, were removed.

We excluded the parameter ‘concomitant use of antithrombotics’ from the analysis of missing clinical data, as participants interpreted this parameter inconsistently, leading to an unreliable estimate.

Data were analysed with descriptive statistics using IBM SPSS Statistics 29.0. Differences in the prevalence of missing and requested data between prescriber type (see secondary outcomes) were analysed with chi-square tests (*p* < 0.05 considered statistically significant).

### Qualitative data from semi-structured interviews

#### Participant sampling and recruitment

Community pharmacists from the Utrecht University region who participated in our quantitative study were randomly selected and invited via telephone to take part in a semi-structured interview. We aimed for an initial sample of eight interviews, based on previous experiences and the focused research question [18]. After these interviews, at least two more interviews were held. Recruitment continued until data saturation, defined as no new codes being identified in two consecutive interviews, was reached [26]. All invited pharmacists were willing to participate and gave their written informed consent before the interview.

#### Data collection

We designed the semi-structured interview guide using the Capability, Opportunity, Motivation, Behaviour (COM-B) system (Online Resource 3) to explore factors influencing pharmacists’ decisions to request additional clinical data [27]. COM-B is a frequently used system for understanding behaviour change in healthcare professions. The interviews started with two different clinical cases of first prescriptions of DOACs, which served as examples for the pharmacists to give more context to the interview questions. The interview guide was piloted with two community pharmacists: one practicing in the Utrecht region and one who had recently stopped practicing. This pilot resulted in minor revisions (e.g. addition of an opening question after the presentation of the cases). Because of the minor revisions, we included the pilot interview of the practicing pharmacist in the Utrecht region.

The semi-structured interviews (conducted in Dutch) were performed by two Master of Pharmacy students (CW and LD) between March and May 2023; either face-to-face or via Microsoft Teams video calls, depending on the participant’s preference. The two student-researchers took alternating roles interviewing or making field notes and asking follow-up questions, if necessary. During the first three interviews, a third researcher (JvP), experienced in qualitative research, was also present to guide and provide feedback to the students. All interviews were audio-recorded. The intended interview time was 25 min.

#### Data analysis

Audio recordings were transcribed verbatim using Amberscript and imported into NVivo (version 20) for analysis. We used the COM-B framework to create a predefined coding tree for deductive analysis (Online Resource 4) [27]. We added any topics that emerged during the coding process by inductive analysis (Online Resource 4). Subsequently we created themes from associated codes.

The first interview was coded separately by the Master students (CW and LD). They checked all selected quotations and codes and discussed similarities and discrepancies. They discussed the discrepancies with the third researcher (JvP) until consensus was reached and a common method of coding was inducted for the subsequent interviews. The subsequent four interviews were coded by CW and LD together and in case of discrepancies again discussed with JvP. The remaining interviews were coded by CW for pragmatic reasons. When in doubt, CW consulted JvP. JvP created the themes, after preliminary work by CW, in consultation with the other researchers.

### Triangulation of quantitative and qualitative data

Finally, quantitative and qualitative data were triangulated through discussion of the findings (reported in the Discussion). In this process, qualitative findings were mainly used to enhance our understanding of the quantitative findings [22, 28]. Specifically, we focused on exploring underlying reasoning that had been elicited via the collection form as part of the quantitative data collection to gain a more comprehensive understanding of pharmacists’ requests for additional data and decision-making when assessing and dispensing first-time DOAC prescriptions.

## Ethics approval

This study was approved by the Institutional Review Board of the Division of Pharmacoepidemiology and Clinical Pharmacology (approval numbers UPF2302: February 10th, 2023, and UPF2305: April 5th, 2023) [29]. Under Dutch law, informed consent was only required and obtained from participating pharmacists, since solely anonymised and non-traceable existing patient prescription data were collected.

## Results

### Quantitative data

**Fig. 1:**
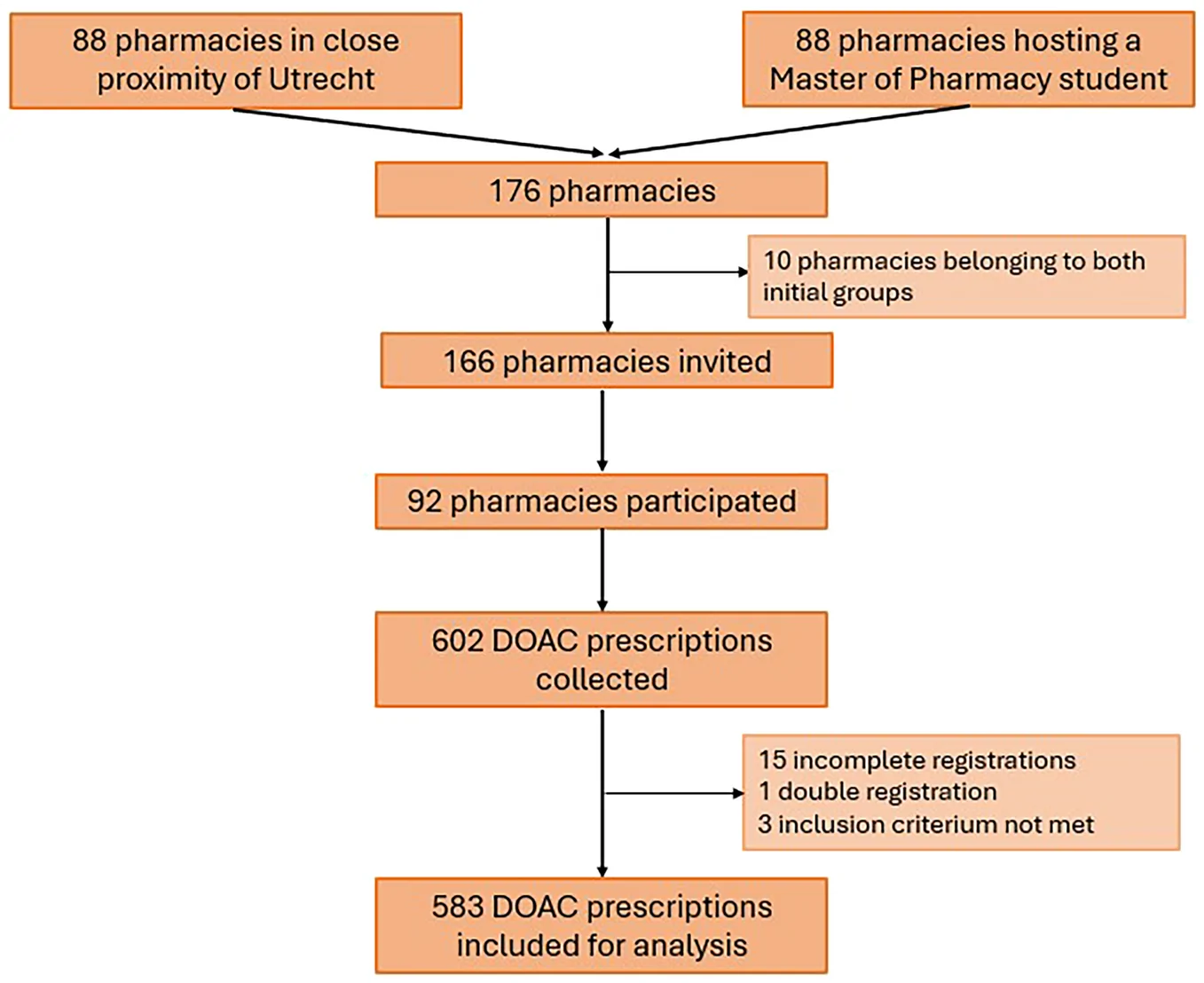
Flowchart of participating pharmacies and collected and included DOAC prescriptions. DOAC = Direct Oral Anticoagulant

In total, 92 participating pharmacies documented 583 eligible first-time DOAC prescriptions (Fig. 1).

The characteristics of these prescriptions are shown in Table 1. The median age of the patients was 74 years (range 18–99), and apixaban (49%; n = 283) and rivaroxaban (38%; n = 220) were most frequently prescribed. AF was the most frequently known indication (21%; n = 120).

**Table 1:** Characteristics of included first-time DOAC prescriptions

Characteristics of prescriptions (n = 583)		Value
Included prescriptions per pharmacy, range		1–29
First-time use of DOAC		503
First-time prescription of DOAC in community pharmacy, but previously dispensed elsewhere		80
	Outpatient pharmacy	30
	Other/unknown	20
	Discharge from hospital	15
	Other community pharmacy	15
Median age patients, years (range; Q1, Q3)		74 (18–99; 64, 81)
Prescriber, n (%)		
	General practitioner	216 (37.0%)
	Cardiologist	161 (27.6%)
	Other ^a^	145 (24.9%)
	Unknown ^b^	61 (10.5%)
DOAC, n (%)		
	Apixaban	283 (48.5%)
	Rivaroxaban	220 (37.7%)
	Edoxaban	56 (9.6%)
	Dabigatran	24 (4.1%)
Indication, n (%)		
	Atrial fibrillation	121 (20.8%)
	Treatment and prevention of VTE	56 (9.6%)
	Prevention VTE after surgery	16 (2.7%)
	Other ^c^	13 (2.2%)
	Thrombophlebitis	4 (0.7%)
	Unknown ^d^	373 (64.0%)

^a^ For example: internist, pulmonologist, or resident; ^b^ Prescriber was not documented on prescription; ^c^ For example: cerebrovascular accident/transient ischemic stroke, or arthritis; ^d^ Indication was not documented on prescription; DOAC = Direct Oral Anticoagulant; VTE = Venous Thromboembolism

**Fig. 2:**
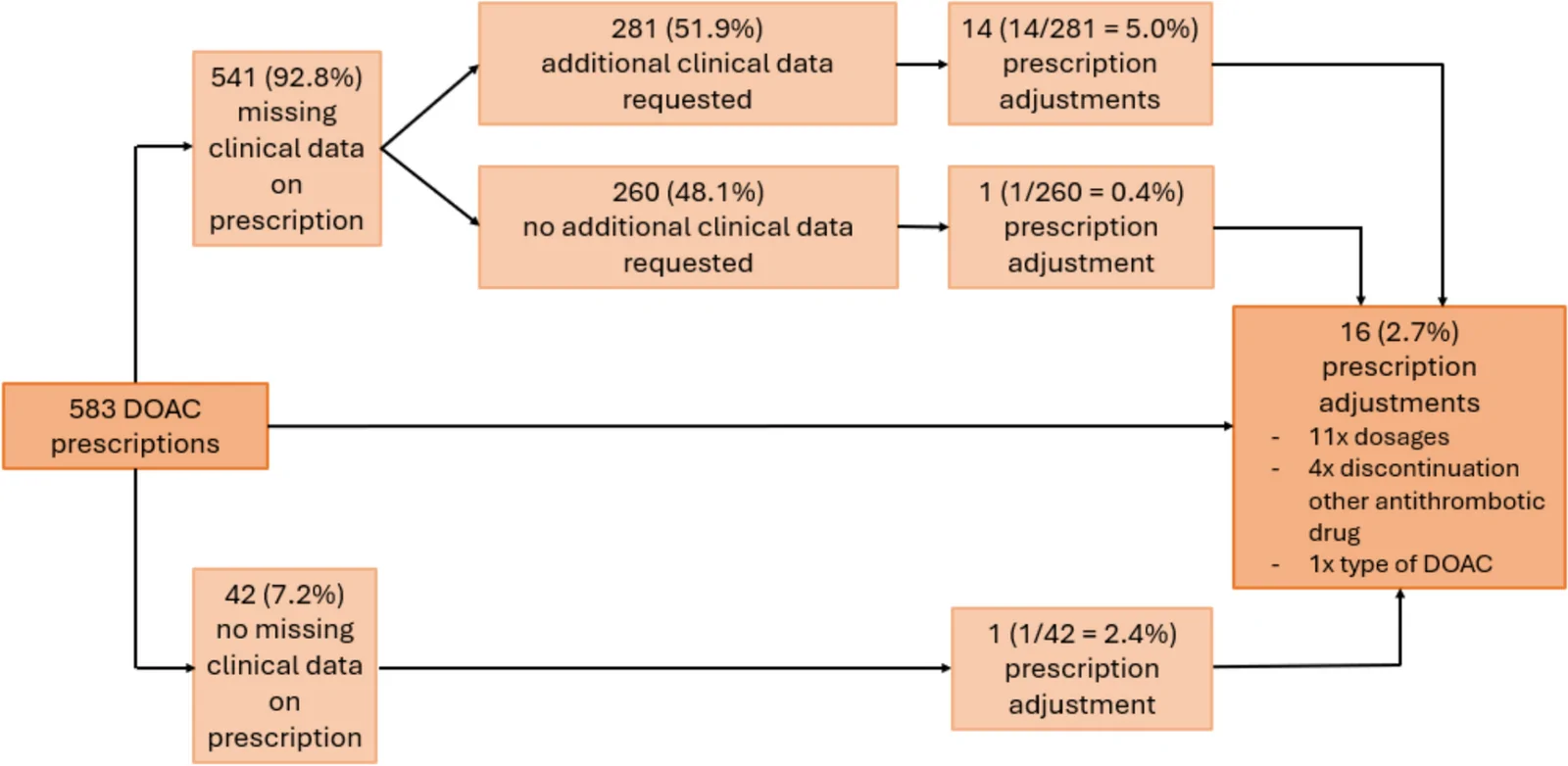
Flowchart of missing and requested clinical data and adjustments of DOAC prescriptions to optimise therapy. Frequencies (n) and percentages (%); DOAC = Direct Oral Anticoagulant

Clinical data were missing in 92.8% (n = 541) of DOAC prescriptions (Fig. 2). Pharmacists requested additional data for 51.9% (n = 281) of prescriptions with missing clinical data, resulting in 14 adjustments among these prescriptions (5.0%). In addition, two prescription adjustments were made without the need for additional data. Overall, pharmacists adjusted 16 of 583 DOAC prescriptions (2.7%): 11 concerned dosage, 4 discontinuation of a concomitant antithrombotic drug, and 1 a change in DOAC type.

#### Missing and requested clinical data

Body weight was most frequently missing on first-time DOAC prescriptions (73.9%; n = 431), followed by indication (64.0%; n = 373). Renal function and treatment duration were missing in 44.8% (n = 261) and 46.3% (n = 270) of prescriptions, respectively (Table 2).

**Table 2:** Missing and requested clinical data for DOAC prescriptions (n = 583)—specified by clinical parameter

		Indication	Renal function	Body weight	Duration of use	Concomitant use of antithrombotics^a^	Other^b^
Missing on prescription (n, (%))		373 (64.0%)	261 (44.8%)	431 (73.9%)	270 (46.3%)	–	NA
Requested clinical data (n, (%))		172 (29.5%)	171 (29.3%)	101 (17.3%)	46 (7.9%)	49 (8.4%)	15 (2.6%)
	Patient (n, (%))	69 (40.1%)	3 (1.8%)	52 (51.5%)	6 (13.0%)	7 (14.3%)	4 (26.7%)
	Prescriber (n, (%))	76 (44.2%)	70 (40.9%)	20 (19.8%)	31 (67.4%)	17 (34.7%)	3 (20.0%)
	System ^c^ (n, (%))	14 (8.1%)	93 (54.4%)	24 (23.8%)	2 (4.3%)	24 (49.0%)	5 (33.3%)
	Other (n, (%))	13 (7.6%)	5 (2.9%)	5 (5.0%)	7 (15.2%)	1 (2.0%)	3 (20.0%)
		*n* = *172*	*n* = *171*	*n* = *101*	*n* = *46*	*n* = *49*	*n* = *15*
Obtained clinical data (n, (%))^d^		146 (84.9%)	139 (81.3%)	86 (85.1%)	21 (45.7%)	43 (87.8%)	14 (93.3%)

^a^Concomitant use of antithrombotics was excluded from the analysis of missing on prescription, as participants interpreted this parameter inconsistently, leading to an unreliable estimate; ^b^ Other could only be requested, not missing on prescription; ^c^ System = pharmacy or general practitioner information system, external lab, or received discharge prescription; ^d^ Numbers and percentages of obtained clinical data are related to requested clinical data; NA = not applicable

Pharmacists most frequently requested indication (29.5%; n = 172) and renal function (29.3%; n = 171). They requested body weight 101 times (17.3%). Despite frequent omission of treatment duration in 46.3% (n = 270) on prescriptions, it was requested in only 7.9% (n = 46). Pharmacists requested information on concomitant use of antithrombotics in 8.4% (n = 49) (Table 2).

Pharmacists obtained > 80% of requested additional clinical data for all clinical parameters, except treatment duration (45.7%). They requested indication of DOAC therapy equally from patients (40.1%; n = 69) and prescribers (44.2%; n = 76). They mainly retrieved renal function via the pharmacy information system or an external laboratory (54.4%; n = 93) and via prescribers (40.9%; n = 70). Pharmacists relied on the prescriber as the primary resource for information on treatment duration (67.4%; n = 31), while they primarily used the pharmacy information system to obtain information on concomitant use of antithrombotics (49.0%; n = 24) (Table 2).

#### Prescriptions from GPs versus medical specialists

Prescriptions from GPs more frequently lacked clinical data compared to those of medical specialists (95.4% vs 90.8%; *p* = 0.049; Online Resource 5). This applied to all clinical data we studied. Requests for additional clinical data occurred more often when the prescription originated from the GP (61.2% vs 45.5%; *p* < 0.001), particularly for indication and renal function. When requesting additional information on concomitant use of antithrombotics, pharmacists consulted medical specialists more often than GPs (20.6% vs 11.1%; *p* = 0.039).

#### Underlying reasoning to request additional clinical data

The pharmacists’ main reason to request additional clinical data for first-time DOAC prescriptions was to evaluate the appropriate dosage (39.6%; n = 231). Pharmacists also frequently cited performing clinical risk management (25.4%; n = 148) and providing patient counselling (18.5%; n = 108) as important reasons. Completing the patient file motivated 19.6% (n = 114) of requests. When no additional clinical data were requested, pharmacists primarily indicated having sufficient information to assess (28.3%; n = 165) and dispense (26.4%; n = 154) the prescription. Only 5.3% (n = 31) mentioned a lack of time as a reason for not requesting additional clinical data.

### Qualitative data

We interviewed 11 community pharmacists in 10 interviews (including the pilot interview). All invited pharmacists participated, meaning a 100% participation rate. In one interview, two pharmacists from the same pharmacy participated. Participants were on average 34 years old (range: 24 to 49 years), 10 were female (1 male), and 4 were managing pharmacists (7 non-managing). Managing pharmacists were pharmacists with managerial responsibilities (e.g., oversight of pharmacy operations and staff), whereas non-managing pharmacists were those without such responsibilities. The mean duration of the interviews was 18 min (range: 11–23 min). In retrospect, data saturation was reached after seven interviews (Online Resource 6).

**Fig. 3:**
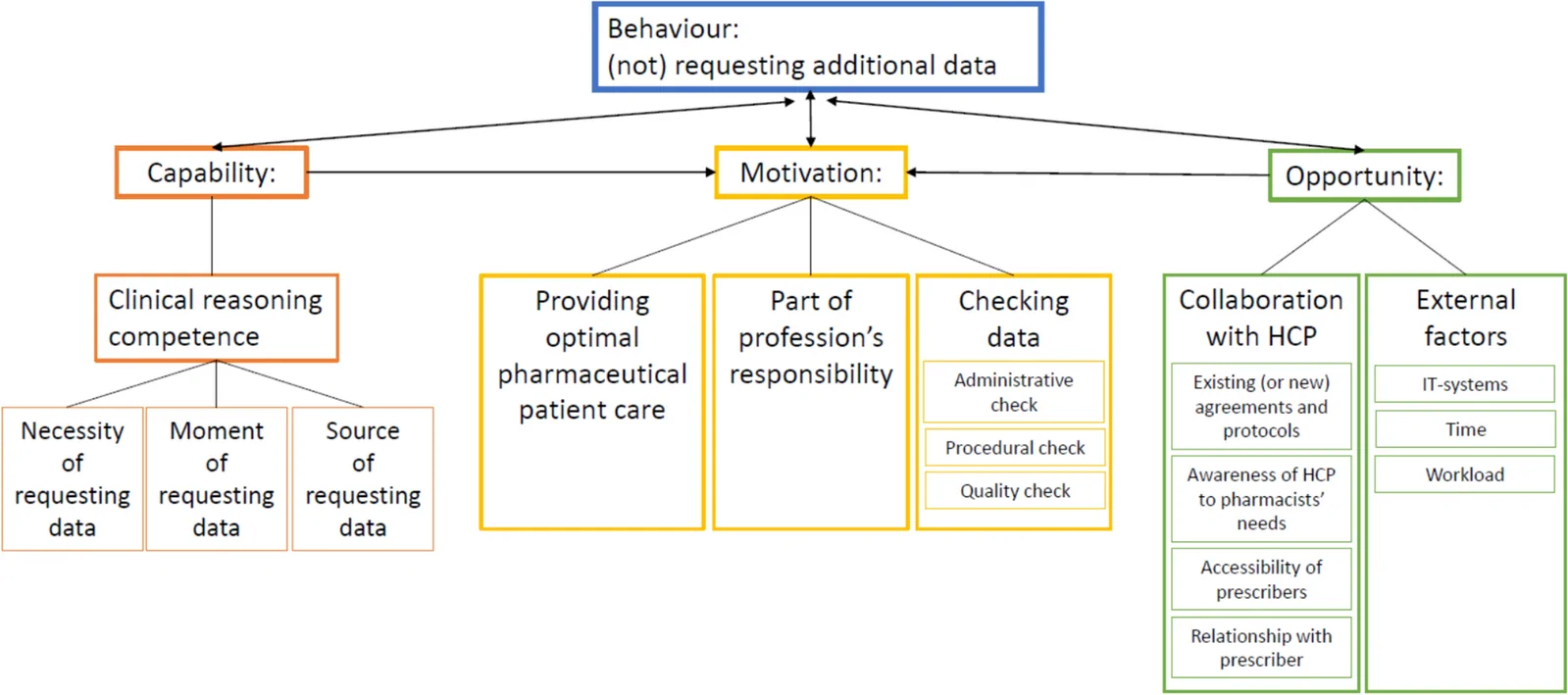
Major themes and their relation to the COM-B system: what prompts the pharmacist to request additional clinical data for DOAC prescriptions. COM-B = Capability Opportunity Motivation – Behaviour; HCP = healthcare professionals

Figure 3 illustrates the major themes of our interviews and their relation to the COM-B system. All three main aspects of the COM-B system – capability, opportunity and motivation – influenced the request for additional clinical data. The associated themes are described below. Exemplifying quotes are listed in Table 3.

**Table 3:** Quotes exemplifying the major themes and associated subthemes of the COM-B system as shown in Fig. 3

COM-B system	Theme and *subtheme*	Quotes
Capability	Clinical reasoning competence	
	*Necessity of requesting data*	"I think pharmacists […] can often do a pretty good risk assessment, including what they know and do not know, but also whether they think something is safe to dispense." Pharmacist 7 – F "If someone is below 80, it is often not very relevant [to ask additional data]. Depends a little bit on which DOAC, you do start asking. It is really: which DOAC is prescribed and which lab values belong to that DOAC and based on that you decide to ask or not to ask additional data." Pharmacist 6 – F
	*Moment of requesting data*	“If a value is at the threshold, I do like a more recent value [before dispensing]. But if it is well above, and it is 1.5 years old, I think it is a different story. That is the consideration, but usually the data is requested immediately.” Pharmacist 4 – F
	*Source of requesting data*	"Look, the patient does not know it's renal function and you will have to request that, but for the indication you can consult the patient and if that is a clear story and also matches the dosage, then I agree." Pharmacist 3 – F
Opportunity	Collaboration with HCPs	
	*Existing (or new) agreements and protocols*	"In our municipality, we did make some agreements already around this process [of indication, renal function and body weight]. So we have also asked the GPs to put this information on the prescription as well to already know more. That does make things easier." Pharmacist 4—F
	*Accessibility of prescribers*	"It is often the medical specialist who prescribes it [DOAC], and if you need further information, you would have to call the hospital. Well, I think that's what every pharmacy experiences sometimes a test of patience and perseverance, so you may need multiple phone calls." Pharmacist 9—M
	*Relationship with prescriber*	"There is an advantage of working in a health centre with the GP under the same roof, having short lines of communication, and the collaboration with our GPs is just, very, very nice and accessible." Pharmacist 2 – F "HCPs are becoming more aware of the importance of providing information to the pharmacy, so some data are starting to appear on the prescription." Pharmacist 4 – F
	External factors	
	*IT-systems*	“We have a connection with the GP, so that when they receive lab data and the patient has given consent, we receive it also, so that helps.” Pharmacist 8 – F
	*Workload and time*	"Time is the biggest limiting factor. Because ideally, of course, you want to figure it [renal function] out in detail for every prescription, but you cannot. So you always have to make a risk assessment. [….] I do notice when it is very busy in the pharmacy, sometimes things slip in, but I do not think that is a reason not to do it." Pharmacist 6 – F
Motivation	Providing optimal pharmaceutical patient care	
	*Providing optimal pharmaceutical patient care*	"On one hand, you want optimal efficacy, but on the other hand, you also want to avoid side effects, particularly bleeding." Pharmacist 3 – F "Having that [indication and renal function] is necessary to do adequate dose evaluation, and without these data you cannot say, this dosage is right, because it really depends on those factors. So I think that is the most important thing to be able to quickly assess: this is appropriate for this patient." Pharmacist 6 – F
	Part of profession’s responsibility	
	*Part of profession’s responsibility*	"Because I want to do dose evaluation properly, otherwise I am only doing half my job. Yes, then I think a robot can also give the medication." Pharmacist 10 – F
	Checking data	
	*Administrative check*	"It is important to have all the data complete afterwards to know whether the dosage is correct, because it often remains chronic medication, to be sure that it is just safe to continue using it that way." Pharmacist 8 – F
	*Procedural check*	"I want the data on the prescription, because we made an agreement for that." Pharmacist 7 – F
	*Quality check*	"We have antithrombotics and double anticoagulation as high-risk medication in our quality system. So we have an action every six months to review this medication. And if you see the medication anyway, reviewing at that moment saves time going through all those patients afterwards." Pharmacist 2 – F

COM-B system = Capability Opportunity Motivation – Behaviour system; HCPs = healthcare professionals; GP = general practitioner

#### Capability

It was important for pharmacists to have sufficient competence in applying clinical reasoning to decide whether additional clinical data was needed. Using clinical reasoning, pharmacists assessed each DOAC prescription and the available clinical data to determine whether to request additional data. The clinical reasoning competence could be divided into three subthemes: the necessity, the moment, and the source of requesting data. First, pharmacists often based this necessity to request additional clinical data on their knowledge of DOACs to perform a risk assessment to derive the required clinical data. Second, pharmacists based the moment of requesting additional clinical data (i.e., prior to dispensing versus after dispensing) on clinical reasoning. Pharmacists decided whether it was safe to dispense the DOAC even without clinical data. Third, pharmacists also involved the source of data needed in their clinical reasoning.

#### Opportunity

Pharmacists experienced several facilitators and barriers that affected their opportunity to request additional clinical data. The main reasons for requesting data could be divided into two important aspects. The first aspect concerned the collaboration with other healthcare professionals (HCPs). Collaboration with GPs was mentioned as a facilitator to support the development of agreements and protocols about sharing relevant clinical data. For pharmacists, the accessibility of prescribers, could function either as a barrier or a facilitator. A strong working relationship with, and easy access to, GPs facilitated the pharmacists’ ability to request additional data. In contrast, limited accessibility to medical specialists working in hospitals, was predominantly perceived as a barrier for requesting additional clinical data. A pharmacist also mentioned a tendency of HCPs becoming more aware of the needs of pharmacists to obtain additional clinical data, which can also be considered collaboration. The second aspect influencing the opportunities of pharmacists requesting additional clinical data concerned the external factors workload, time, and IT-systems. IT-systems could be perceived as both a facilitator and a barrier. On one hand, these IT-systems could enhance access to external data from other HCPs or clinical laboratories; on the other hand, they could also restrict the available information on a prescription, for example, in the case of a repeat prescription. Pharmacists commonly identified workload and limited time as barriers for requesting additional clinical data.

#### Motivation

All pharmacists cited DOAC dose evaluation as underlying reasons to request additional clinical data. Some pharmacists also cited clinical risk management, patient counselling and the completion of the pharmaceutical patient file as reasons to request data. Pharmacists regarded these reasons as important, based on three main motivations. First, all pharmacists emphasized their commitment to providing optimal pharmaceutical patient care as a key driver for requesting additional clinical data. Second, pharmacists viewed all underlying reasons to request additional clinical data, particularly dose evaluation, as an integral part of their profession’s responsibility. Third, pharmacists cited the importance of data verification as a motivation to request additional clinical data. These checks could serve various purposes: administrative (ensuring a complete pharmaceutical patient file), procedural (adhering to established agreements), or quality-related (upholding professional standards).

## Discussion

This study showed that clinical data were frequently missing when first-time DOAC prescriptions were presented in community pharmacies. As a consequence, pharmacists requested additional clinical data, particularly indication and renal function, in 52% of these prescriptions. Ultimately this resulted in adjustments of 5% of these prescriptions for which additional clinical data were requested. Pharmacists applied clinical reasoning to determine whether additional data was needed. The opportunity to request data was influenced by the collaboration with other HCPs and by external factors such as IT-systems, time constraints, and workload. Workload was cited as a reason for not requesting missing information by only 5% of pharmacists. The pharmacists’ primary motivation to request additional data was to optimise a patient’s treatment by appropriate dosing.

Of all our collected first-time DOAC prescriptions (n = 583), 2.7% was adjusted. A previous study in the Netherlands showed that overall 1.8% of prescriptions in the community pharmacy were modified to solve or prevent a drug related problem [30]. This suggests that first-time DOAC prescriptions relatively often lead to prescription changes. Inappropriate DOAC prescriptions could lead to a suboptimal balance between thrombotic- and bleeding complications for the patient, with an unfavourable clinical outcome [1, 6]. Reviewing DOAC prescriptions is therefore an important responsibility in optimising DOAC therapy, as supported by both our quantitative and qualitative findings. Pharmacists requested additional clinical data for more than half of prescriptions and identified optimising patient treatment as their primary motivation in our interviews and based on our quantitative data, indicating 39.6% requested data to evaluate appropriate dosage. When pharmacists did not request additional clinical data, they primarily indicated in our quantitative data having sufficient data to assess or dispense the prescription, a finding aligned with interview data indicating reliance on clinical reasoning.

Most prescribing errors for DOACs are dose-related [5]. Based on both our quantitative and qualitative data, the primary motivational reason for requesting additional clinical data was to verify correct dosing. To perform adequate dose evaluation and reduce dose-related errors, pharmacists may require additional clinical data [16–18]. Dose adjustment criteria for DOACs include treatment indication, renal function, body weight, and age, varying per DOAC and complicating dosing decisions [4]. Studies identify treatment indication and renal function as main drivers of inappropriate dosing [6, 14, 31, 32]. Missing these clinical data hampers pharmacists in performing their role in antithrombotic care, despite evidence that their interventions improve appropriate antithrombotic therapy [33]. Our findings supported the pharmacists’ role in verifying dose adjustment criteria and in applying clinical reasoning to do so. To assess dose adjustment criteria properly, clinical reasoning and knowledge of antithrombotics are seen as key components of clinical decision making [18, 34].

Besides data availability, good collaboration with other HCPs had also been mentioned as an opportunity to improve pharmaceutical care provision in general and specifically in antithrombotic care, including in this and earlier studies [18, 34–36]. A multidisciplinary approach and smooth coordination between primary and secondary care to provide optimal treatment is also highlighted in the European Society of Cardiology guideline on AF-management as the golden standard [1]. The qualitative part of our study suggested that closer collaboration with GPs made them more approachable. Therefore pharmacists requested additional clinical data more frequently for prescriptions originating from GPs than for those from medical specialists (61.2% vs 45.5%). These medical specialists (in the hospital) are generally more difficult to contact [18, 34], which is a barrier for requesting additional clinical data. In a Dutch study on adoption of antithrombotic stewardship in hospitals, care transition optimisation is not well implemented yet in the majority of participating hospitals, which is supported by our findings [37].

Clinical data sharing, interprofessional collaboration and pharmacists’ professional responsibility were also identified in a recent study as key barriers and enablers of deprescribing [35]. These factors could therefore be considered crucial for optimising the pharmacists’ role across various forms of pharmaceutical patient care.

### Strengths and limitations

The major strength of this study lied in the large number of prescriptions we collected from daily clinical practice across multiple settings. By using a standardized data collection form, including free text fields, we enhanced data reliability and provided a reliable perspective on missing data on first-time DOAC prescriptions and the opportunity to improve DOAC prescribing. Combining these quantitative data with in-depth interviews, reinforced this perception.

The quantitative part of the study had several limitations. One limitation was a potential overestimation by selective reporting of incomplete DOAC prescriptions. Participating community pharmacists might have been more vigilant when assessing first-time DOAC prescriptions and might have included more delayed requests for additional clinical data, potentially leading to more adjustments. Data collection by master students might better represent non-participating pharmacists, thereby improving the generalisability of our results. Another limitation was the possibility of performance bias, as participants might have gained knowledge during the collection period and altered their assessment behaviour, simply by taking part in the study. Again, the students served as a useful counterbalance, as their supervising pharmacists assessed the DOAC prescriptions. To address these limitations, we analysed data from pharmacists and students separately and found similar results, supporting the reliability of our findings.

Our definition of first-time DOAC prescription might have resulted in misclassifying continuation therapy as new treatment. We assumed this did not affect the presence of missing clinical data on prescriptions. However, it might have reduced the observed number of prescription adjustments, as some prescriptions could have been previously optimised in other pharmacies.

The qualitative part of our study also had limitations. We conducted the interviews along the data collection using random sampling. Purposive sampling might have been more adequate to identify the number of prescriptions collected as a relevant characteristic. In retrospect, however, a well-distributed variation was achieved among participating pharmacists in the collected prescriptions (range 4–29).

The student researchers were previously trained in communication skills but received no specific training in qualitative interviewing, which might have influenced the richness of our findings.

Finally, only pharmacists who already participated in our quantitative study were interviewed, resulting in a potentially non-homogeneous interview sample [26]. The qualitative findings therefore solely reflect the perceptions of pharmacists who were willing to participate.

### Suggestions for clinical practice and future research

This study emphasises the importance of a good collaboration between HCPs, including community pharmacists, to optimise DOAC prescribing by sharing clinical data. DOAC prescribers should be more aware of the importance of sharing clinical data with other HCPs to enhance safe and effective use of DOACs. Local or regional agreements between primary and/or secondary care HCPs on sharing these data by including it on the prescription for example, should get greater emphasis. Regularly reviewing these agreements should be an important precondition. To enhance effective collaboration and agreements, future research could explore the perspectives and needs of all HCPs regarding clinical data sharing.

Another suggestion is to optimise DOAC prescribing skills to reduce inappropriate prescriptions. An international study showed young doctors lack knowledge on prescribing anticoagulants [38]. More intensive education by pharmacists during their studies and after their graduation could be a suitable intervention.

## Conclusion

This study shows community pharmacists frequently request missing clinical data, particularly treatment indication and renal function, to verify DOAC prescriptions and to ensure optimal patient care by using their clinical reasoning competence. Clinical data sharing could be optimised by streamlined collaboration between healthcare professionals.

## Supplementary Information

Below is the link to the electronic supplementary material.

Supplementary file 1 (PDF 167 KB)

Supplementary file 2 (PDF 189 KB)

Supplementary file 3 (PDF 112 KB)

Supplementary file 4 (PDF 154 KB)

Supplementary file 5 (PDF 113 KB)

Supplementary file 6 (PDF 99 KB)

## Data Availability

The quantitative data that support the findings of this study are available from the corresponding author upon reasonable request. For the qualitative data that support the findings of this study we can only share the analysis upon reasonable request.
