# Supplementary material for: Access to clinical data for direct oral anticoagulant prescriptions in community pharmacy and implications for medication optimisation: a mixed-methods study
Source: Int J Clin Pharm. 2026 May 22;48(5):1871–83. doi: 10.1007/s11096-026-02159-3 (PMC13569597; doi:10.1007/s11096-026-02159-3)
Supplement: Supplementary file 1 [file 11096_2026_2159_MOESM1_ESM.pdf]

Supplementary information:

Online Resource 1: COREQ – checklist [1]:

Domain 1: research team and reflexivity		
**Personal characteristics**		**Answer**
1. Interviewer/facilitator	Which author/s conducted the interview or focus group?	Two student-researchers (CW and LD) with help from Jacqueline van Paassen (JvP)
2. Credentials	What were the researcher's credentials?	CW and LD: BSc, Master of Pharmacy students<br>JvP: PharmD, PhD-candidate<br>VD: PhD, PharmD<br>TK: PhD, PharmD → joined the study in the analysis phase<br>MB: PhD, PharmD
3. Occupation	What was their occupation at the time of the study?	CW and LD: Master of Pharmacy students<br>JvP: PhD-candidate and Lecturer at Utrecht University (history of 18 years of work as a community pharmacist)<br>VD: hospital pharmacist and associate professor<br>TK: pharmacist and associate professor<br>MB: pharmacist and professor
4. Gender	Was the researcher male or female?	CW and LD: female<br>JvP, VD: female<br>TK, MB: male
5. Experience and training	What experience or training did the researcher have?	CW and LD: general communication training during their Pharmacy education<br>JvP: 18 years of work as a community pharmacist. Lecturer in oral communication skills and equipped with a qualitative interview course<br>VD: >20 years as a hospital pharmacist – clinical pharmacologist, >20 years in research<br>TK: >10 years as clinical pharmacist, >10 years in research<br>MB: >30 years as a community pharmacist, >20 years in research
**Relationship with participants**		
6. Relationship established	Was a relationship established prior to study commencement?	Pharmacists who participated in the qualitative study were already enrolled in the quantitative study of this mixed-methods study

7. Participant knowledge of the interviewer	What did the participants know about the researcher?	The pharmacists knew the name of JvP, VD and MB when assigning to participate
8. Interviewer characteristics	What characteristics were reported about the interviewer / facilitator? e.g. bias, assumptions, reasons and interests in the research topic	JvP is a former community pharmacist. CW and LD are Master student-researchers
Domain 2: study design		
**Theoretical framework**		
9. Methodological orientation and theory	What methodological orientation was stated to underpin the study? E.g. grounded theory, discourse analysis, ethnography, phenomenology, content analysis	The Capability, Opportunity, Motivation, Behaviour (COM-B) system
**Participant selection**		
10. Sampling	How were participants selected? e.g. purposive, convenience, consecutive, snowball	Pharmacists who participated in the quantitative study were randomly selected and invited.<br>For the pilot interviews convenience sampling was used.
11. Method of approach	How were participants approached? e.g. face-to-face, telephone, mail, email	The participants were approached via telephone.
12. Sample size	How many participants were in the study?	11 participants in 10 interviews (including the pilot interview)
13. Non-participation	How many people refused to participate or dropped out? Reasons?	0
**Setting**		
14. Setting of data collection	Where was the data collected? e.g. home, clinic, workplace	Via MS Teams or face-to-face at the community pharmacy.
15. Presence of non-participants	Was anyone else present besides the participants and researchers?	No
16. Description of sample	What are the important characteristics of the sample? e.g. demographic data, date	Participants were on average 34 years old (range: 24 to 49 years), 10 were female (1 male), and 4 were managing pharmacists (7 non-managing).
**Data collection**		
17. Interview guide	Were questions, prompts, guides provided by the authors? Was it pilot tested?	The researchers used an interview protocol which was pilot tested in advance.<br>The participants did not receive the interview protocol in advance. They

		did receive information on the objective of the study.
18. Repeat interviews	Were repeat interviews carried out? If yes, how many?	No
19. Audio/visual recording	Did the research use audio or visual recording to collect the data?	Audio recording was used to collect the data and to transcribe the interview.
20. Field notes	Were field notes made during and/or after the interview or focus group?	Yes, made during the interview by the secondary interviewer and complemented after the interview by the interviewer.
21. Duration	What was the duration of the interviews or focus group?	Mean duration 18 min (range: 11-23 min)
22. Data saturation	Was data saturation discussed?	Yes
23. Transcripts returned	Were transcripts returned to participants for comment and/or correction?	The participants were given the option to comment on their transcript; ultimately, no transcripts were returned.
Domain 3: analysis and findings		
**Data analysis**		
24. Number of data coders	How many data coders coded the data?	The first interview was coded separately by the two students (CW and LD). They checked all selected citations and codes and discussed similarities and discrepancies. The discrepancies were then discussed with the third researcher (JvP) until consensus was reached and a common method of coding was inducted for the following interviews. The following 4 interviews were coded by the 2 students together and in case of discrepancies again discussed with the third researcher. The remaining interviews were coded by one of the students (CW) for pragmatic reasons. When in doubt, CW consulted the third researcher (JvP). JvP created the themes, after preliminary work by CW.
25. Description of the coding tree	Did authors provide a description of the coding tree?	Yes, in the supplementary material
26. Derivation of themes	Were themes identified in advance or derived from the data?	Codes were identified in advance. We made use of open-coding in case new codes derived.<br>Overarching themes and subthemes were derived by analysing and interpreting the codes. All researchers were involved in creating the themes.

27. Software	What software, if applicable, was used to manage the data?	NVivo version 20
28. Participant checking	Did participants provide feedback on the findings?	The participants receive a summary of the findings after publication.
**Reporting**		
29. Quotations presented	Were participant quotations presented to illustrate the themes/findings? Was each quotation identified? e.g. participant number	Yes
30. Data and findings consistent	Was there consistency between the data presented and the findings?	Yes
31. Clarity of major themes	Were major themes clearly presented in the findings?	Yes
32. Clarity of minor themes	Is there a description of diverse cases or discussion of minor themes?	Yes

<u>Reference:</u>

[1] Tong A, Sainsbury P, Craig J. Consolidated criteria for reporting qualitative research (COREQ): A 32-item checklist for interviews and focus groups. Int J Qual Health Care 2007;19:349–57. https://doi.org/10.1093/intqhc/mzm042.
