# Supplementary material for: Access to clinical data for direct oral anticoagulant prescriptions in community pharmacy and implications for medication optimisation: a mixed-methods study
Source: Int J Clin Pharm. 2026 May 22;48(5):1871–83. doi: 10.1007/s11096-026-02159-3 (PMC13569597; doi:10.1007/s11096-026-02159-3)
Supplement: Supplementary file 2 [file 11096_2026_2159_MOESM2_ESM.pdf]

Supplementary information:

Online resource 2: Registration form (translated from Dutch)

Study code (pharmacy code + date dispensing prescription ddmmyy + sequence letter):

General data:		
Prescription:	Which prescription is offered at the pharmacy?<br>☐ Regular (outpatient) prescription<br>☐ Discharge prescription form hospital<br>☐ Repeat prescription from outpatient pharmacy<br>☐ Repeat prescription from other community pharmacy<br><br>Is the patient going to use the DOAC for the first time?<br>☐ Yes<br>☐ No<br><br>If no, where was the first DOAC prescription dispensed?<br>☐ Outpatient pharmacy<br>☐ Other community pharmacy<br>☐ During hospital admission (considered discharge prescription)<br>☐ Other: …………………<br>☐ Unknown	
Patient data:		
Gender:	☐ Male<br>☐ Female<br>☐ X	
Year of birth (4 digits):	…………….	
Prescription data:	***Information PRESENT on prescription***	***Information ABSENT on prescription***
Prescribing physician:	☐ General Practitioner<br>☐ Cardiologist<br>☐ Orthopaedic surgeon<br>☐ Internist<br>☐ Pulmonologist<br>☐ Neurologist<br>☐ Other: …………………..<br><br>If originating from medical specialists, from which hospital:<br>☐ Regional hospital 1<br>☐ Regional hospital 2<br>☐ Regional hospital 3<br>☐ Other: …………………….<br><br>Is the name of the prescriber known?<br>(in case of repeat prescription: the initial prescriber)<br>☐ Yes	☐ Prescribing physician<br><br>☐ Hospital<br><br>☐ Name unknown
DOAC:	☐ Apixaban (Eliquis of generic)	☐ DOAC

	☐ Dabigatran (Pradaxa)<br>☐ Edoxaban (Lixiana)<br>☐ Rivaroxaban (Xarelto)	
Clinical data:	***Information PRESENT on prescription:***	***Information ABSENT on prescription:***
Strength (mg) and daily dose:	……….. mg<br>………… time(s) per day	☐ Strength<br>☐ Daily dose
Body weight patient (kg):	……………. kg	☐ Body weight
Intended duration of use:	☐ Chronic<br>☐ Short term: ………………… weeks / months / year	☐ Duration of use
Indication:	☐ Atrial fibrillation<br>☐ Treatment and prevention of recurrent deep vein thrombosis or pulmonary embolism<br>☐ Prophylaxis venous thromboembolism after surgery<br>☐ Thrombophlebitis<br>☐ Other: ………………………	☐ Indication
Renal function (eGFR) patient:	……………. ml/min/1,73m^2<br>☐ CKD-EPI<br>☐ MDRD<br>☐ Cockcroft Gault<br><br>Measurement date eGFR: ……/……/……<br>Serum creatinine: ……….. µmol/l	☐ eGFR<br>☐ Measurement method<br><br>☐ Measurement date<br>☐ Serum creatinine
Combination of antithrombotics:	Is the DOAC combined with another antithrombotic?<br>☐ Yes<br>☐ No<br><br>If yes, other antithrombotic:<br>☐ Acetylsalicylic acid<br>☐ Carbasalate calcium<br>☐ Clopidogrel<br>☐ Prasugrel (Efient)<br>☐ Ticagrelor (Brilique)<br>☐ LMWH<br>☐ Other: …………………<br><br>Duration of use other antithrombotic:<br>☐ Chronic<br>☐ Short term: ………………… weeks / months / year	☐ Combination unknown<br><br>☐ Other antithrombotic<br><br>☐ Duration of use other antithrombotic
Dispensing information:		

- No information absent, go to the end of the form.
- If any information was absent in the above questions, please answer the questions below:

Prescription and/or clinical data are incomplete. Will you take any further action?

☐ Yes
☐ No

If yes, why do you take action on the incomplete data on this first DOAC prescription? (if multiple answers, number them in order of importance, where 1 = most important)

☐ Obtain information for clinical risk management (interaction/contra indication/double medication/…)
☐ Obtain information for dose evaluation

- ☐ Obtain information for patient counselling
- ☐ Obtain information to complete patient file
- ☐ Other: ………………

If no, no further action because: (multiple answers possible)

- ☐ Sufficient information for clinical risk management / dosage control / clinical reasoning / patient counselling)
- ☐ Sufficient information for dispensing prescription
- ☐ No time to request additional information
- ☐ Other: …………….

***If further action*:** which data are additionally requested? (multiple answers possible)

- ☐ Body weight
- ☐ Renal function values (eGFR, creatinine)
- ☐ Indication
- ☐ Intended duration of use
- ☐ Combination of antithrombotics
- ☐ Other: ……………….

From whom are these data requested? (multiple answers possible; indicate per requested item)

- ☐ Patient
- ☐ (Assistant of) prescriber of (original) prescription
- ☐ General Practitioner (if not prescriber)
- ☐ Pharmacy Information System
- ☐ External laboratory
- ☐ Other: …………………

Outcome of requested data:

- ☐ Received (including value): ……………………………………
- ☐ Not received: ……………………………………
- ☐ Unknown at source ……………………………………

What was the benefit of obtaining additional data? (multiple answers possible)

- ☐ Additional information to proceed with dispensing
- ☐ Complete patient file
- ☐ Complete clinical risk management / dosage control
- ☐ Providing good patient counselling
- ☐ Other: …………………..

Did the additional data lead to adjustment of the prescription?

- ☐ Yes
- ☐ No
- ☐ No additional information was received. Comment: ……………………………….

And if yes, which adjustment? (multiple answers possible)

- ☐ Change DOAC to: ………………………………….
- ☐ Stop DOAC
- ☐ Change strength (mg) to: ……………………
- ☐ Change daily dose (number of times per day) to: ………………..
- ☐ Change duration of use to: ……………………………
- ☐ Add duration of use to: …………………………………
- ☐ Change other antithrombotic to: …………………………….
- ☐ Discontinue other antithrombotic
- ☐ Other: ……………………………………………………………………

Was the adjustment made in consultation with the prescriber?

- ☐ Yes
- ☐ No

How much time did it take to dispense (including control) the DOAC prescription

- ☐ < 5 min
- ☐ 5-15 min

- ☐ 15-30 min
- ☐ 30-45 min
- ☐ 45-60 min
- ☐ >60 min

Any other comments:
……
……

You have finished filling out this form

When no information was absent or no information additionally requested in the initial situation:

Did the existing (present) information lead to adjustment of the prescription?

- ☐ Yes
- ☐ No

And if yes, which adjustment? (multiple answers possible)

- ☐ Change DOAC to: ………………………………….
- ☐ Stop DOAC
- ☐ Change strength (mg) to: ……………………
- ☐ Change daily dose (number of times per day) to: ……………….
- ☐ Change duration of use to: …………………………….
- ☐ Add duration of use to: …………………………………
- ☐ Change other antithrombotic to: ……………………………..
- ☐ Discontinue other antithrombotic
- ☐ Other: ………………………………………………………………………

Was the adjustment made in consultation with the prescriber?

- ☐ Yes
- ☐ No

How much time did it take to dispense (including control) the DOAC prescription

- ☐ < 5 min
- ☐ 5-15 min
- ☐ 15-30 min
- ☐ 30-45 min
- ☐ 45-60 min
- ☐ >60 min

Any other comments:
……
……

You have finished filling out this form
