# Supplementary material for: Access to clinical data for direct oral anticoagulant prescriptions in community pharmacy and implications for medication optimisation: a mixed-methods study
Source: Int J Clin Pharm. 2026 May 22;48(5):1871–83. doi: 10.1007/s11096-026-02159-3 (PMC13569597; doi:10.1007/s11096-026-02159-3)
Supplement: Supplementary file 3 [file 11096_2026_2159_MOESM3_ESM.pdf]

Supplementary information:

Online Resource 3: interview protocol (translated from Dutch)

Interview protocol:

Introduction:
Good morning/good afternoon,
First of all, thank you for the opportunity to interview you about the assessing and dispensing of first DOAC prescriptions. As you know, for the EU DOAC study, information is being collected on the data present on the first present of a DOAC and also on your perceptions for requesting missing information. With this interview, we would like to find out why you want certain data on an first DOAC prescription. Here, we are curious about the added value of this data and what drives you to request it. Furthermore, we would like to know the barriers for obtaining data and the opportunities to improve its availability and therefore to optimise patient care in the future. This interview will not test for knowledge, it is really about your opinion.

The interview will be divided into a number of topics. I would like to ask you some questions about your opinion, your experience and about your vision for the future. At the end, there will be an opportunity to raise a topic of your own if you think we are missing something.
The interview will last about 20 to 25 minutes in total. Do you have any questions for me so far?

'I will now start the recording.'

I would like to start by presenting two cases. Using these cases as examples will make it easier to put the following questions into context. You don't have to stick to them when answering the questions, they really serve as examples.

Case study 1:
On Friday afternoon, Annie (62 years old) enters the pharmacy. She has just come from the GP and needs to start apixaban 10 mg twice a day for 7 days, which will become 5 mg twice a day after that week. She has had asthma all her life. For her asthma, she takes the following medication:
- Salbutamol inhaler 100 micrograms, if needed, maximum 8 times daily;
- Salmeterol/fluticasone disk inhaler 50/100 micrograms, 2 times a day 1 inhalation.

Case study 2:
On Friday afternoon, Linda (81 years old) enters the pharmacy. She has just come from the GP and needs to stop her acenocoumarol and start apixaban 5 mg twice a day. Linda has several comorbidities. She has hypertension, hypercholesterolemia, heart failure and diabetes. For these, she takes the following medication:
- Simvastatin 40 mg, once daily
- Candesartan 16 mg, once daily
- Furosemide 20 mg, twice daily
- Metoprolol retard 100 mg, once daily
- Metformin 1000 mg, three times a day
- Gliclazide 60 mg. once daily
- Humulin (insulin), as per doctor's prescription

Questions:

1. Which data do you consider most important to assess a first DOAC prescription?
   a. Why do you think it is important?
2. What are your considerations/reasons to request more data for a first DOAC prescription?
   a. Which consideration/reason is most important to you and why?
3. What are considerations/reasons for you not to request additional data with a first DOAC prescription?
   a. Which consideration/reason is most important to you and why?
4. What are barriers to requesting additional data? (Collaborations, working protocols, time)
   a. What makes this a barrier?
5. What factors make it easy for you to request additional data?
   a. What makes it easier for you?
6. What opportunities do you see to improve the processing of a first DOAC prescription?
   a. What is your role in this matter?
   b. What do you need for this opportunity to happen?

7. End: These were all our questions. Is there anything we have not discussed yet that you would like to add?
