# Supplementary material for: Access to clinical data for direct oral anticoagulant prescriptions in community pharmacy and implications for medication optimisation: a mixed-methods study
Source: Int J Clin Pharm. 2026 May 22;48(5):1871–83. doi: 10.1007/s11096-026-02159-3 (PMC13569597; doi:10.1007/s11096-026-02159-3)
Supplement: Supplementary file 4 [file 11096_2026_2159_MOESM4_ESM.pdf]

Supplementary information:

Online Resource 4: coding tree

Legend:
Normal text: predefined coding tree
Italic: new (inductive) code
Blue: code name changed
~~Strike through~~: unused code

* GP = General Practitioner

Capability

- Psychological capability
    - Knowledge about DOACs
    - Clinical reasoning
        - *Requesting data*
        - *Data source*
        - *Moment of requesting data*

Opportunity

- Physical opportunity
    - Facilitators
        - Systems for data access
        - Work protocols
        - *Contact with GP* for additional information*
        - *Agreements*
        - *Awareness*
        - *Data available on prescription*
        - *Reminder pharmacy information system for evaluating treatment*
    - Limitations
        - Systems for data access
        - Amount of staff
        - Time
        - High workload
        - *Human mistakes*
        - *Communication with prescribers*
        - *Accessibility of prescribers*
        - *Handover of patient treatment*
        - *Repeat prescription as first prescription in the pharmacy*
        - *Undetermined treatment duration*
    - Opportunities
        - System for data availability
        - Obligatory information on prescription
        - Role in opportunities
        - *Handover of patient treatment*
        - New agreements
            - ~~AVMN (regional pharmacy cooperation)~~
- Social opportunity
    - Close connections with prescribers
    - *Requesting sensitive personal information*

Motivation

- Reflective motivation
  - *Willingness of healthcare provider to change prescription*
  - *Reasons for not requesting data*
    - *Complete prescription*
    - *Correct prescription*
    - *Patient counselling*
  - Reasons for requesting data
    - Pharmaceutical patient care
    - Part of profession
    - Prescription based
    - *Clinical risk management and patient counselling*
    - *Complete patient file*
    - *Dose evaluation*
    - *Quality requirements*
    - *Sticking to agreement*
    - *Consequences for pharmacist*
  - ~~Limitations for improvement~~
    - ~~Compensation~~
    - ~~Knowledge~~
  - ~~Opportunities for improvement~~
    - ~~Compensation~~
    - ~~Knowledge~~
    - ~~Systems~~
    - ~~Pharmacy team~~
- Automatic motivation
  - Communication with prescribers
    - ~~Hospital~~
    - ~~General practitioner~~
    - ~~Unspecified prescriber~~
