# Supplementary material for: Access to clinical data for direct oral anticoagulant prescriptions in community pharmacy and implications for medication optimisation: a mixed-methods study
Source: Int J Clin Pharm. 2026 May 22;48(5):1871–83. doi: 10.1007/s11096-026-02159-3 (PMC13569597; doi:10.1007/s11096-026-02159-3)
Supplement: Supplementary file 5 [file 11096_2026_2159_MOESM5_ESM.pdf]

Supplementary information:

Online Resource 5: table with differences in lacking and requested clinical data for prescription of general practitioners versus medical specialists

Table online resource 5: Differences in missing and requested clinical data for prescription of general practitioners versus medical specialists

		Prescriptions from GP (n = 216)	*Prescriptions from medical specialists (n = 305)*	*p-value* *
Clinical data missing on prescription		206 (95.4%)	277 (90.8%)	**0.049**
	Treatment indication	155 (71.8%)	170 (55.7%)	**<0.001**
	Renal function	144 (66.7%)	82 (26.9%)	**<0.001**
	Body weight	192 (88.9%)	188 61.6%)	**<0.001**
	Duration of use	126 (58.3%)	108 (35.4%)	**<0.001**
		GP (n = 206)	*Hospital (n = 277)*	*p-value* *
Requested clinical data when missing on prescription		126 (61.2%)	126 (45.5%)	**<0.001**
		GP (n = 126)	*Hospital (n = 126)*	*p-value* *
	Treatment indication	91 (72.2%)	64 (50.8%)	**<0.001**
	Renal function	99 (78.6%)	56 (44.4%)	**<0.001**
	Body weight	48 (38.1%)	44 (34.9%)	0.601
	Duration of use	25 (19.8%)	14 (11.1%)	0.055
	Combination of antithrombotics	14 (11.1%)	26 (20.6%)	**0.039**

* derived from Chi-square test (bold = statistically significant ($p < 0.05$)); GP = general practitioner
