# Supplementary material for: Access to clinical data for direct oral anticoagulant prescriptions in community pharmacy and implications for medication optimisation: a mixed-methods study
Source: Int J Clin Pharm. 2026 May 22;48(5):1871–83. doi: 10.1007/s11096-026-02159-3 (PMC13569597; doi:10.1007/s11096-026-02159-3)

Access to clinical data for direct oral anticoagulant prescriptions in community pharmacy and implications for medication optimisation: A mixed-methods study – Int J Clin Pharm – JG van Paassen, VHM Deneer, TGH Kempen, ML Bouvy – Utrecht Institute of Pharmaceutical Sciences, Utrecht University, The Netherlands – j.g.vanpaassen@uu.nl

Supplementary information:

Online Resource 6: data saturation

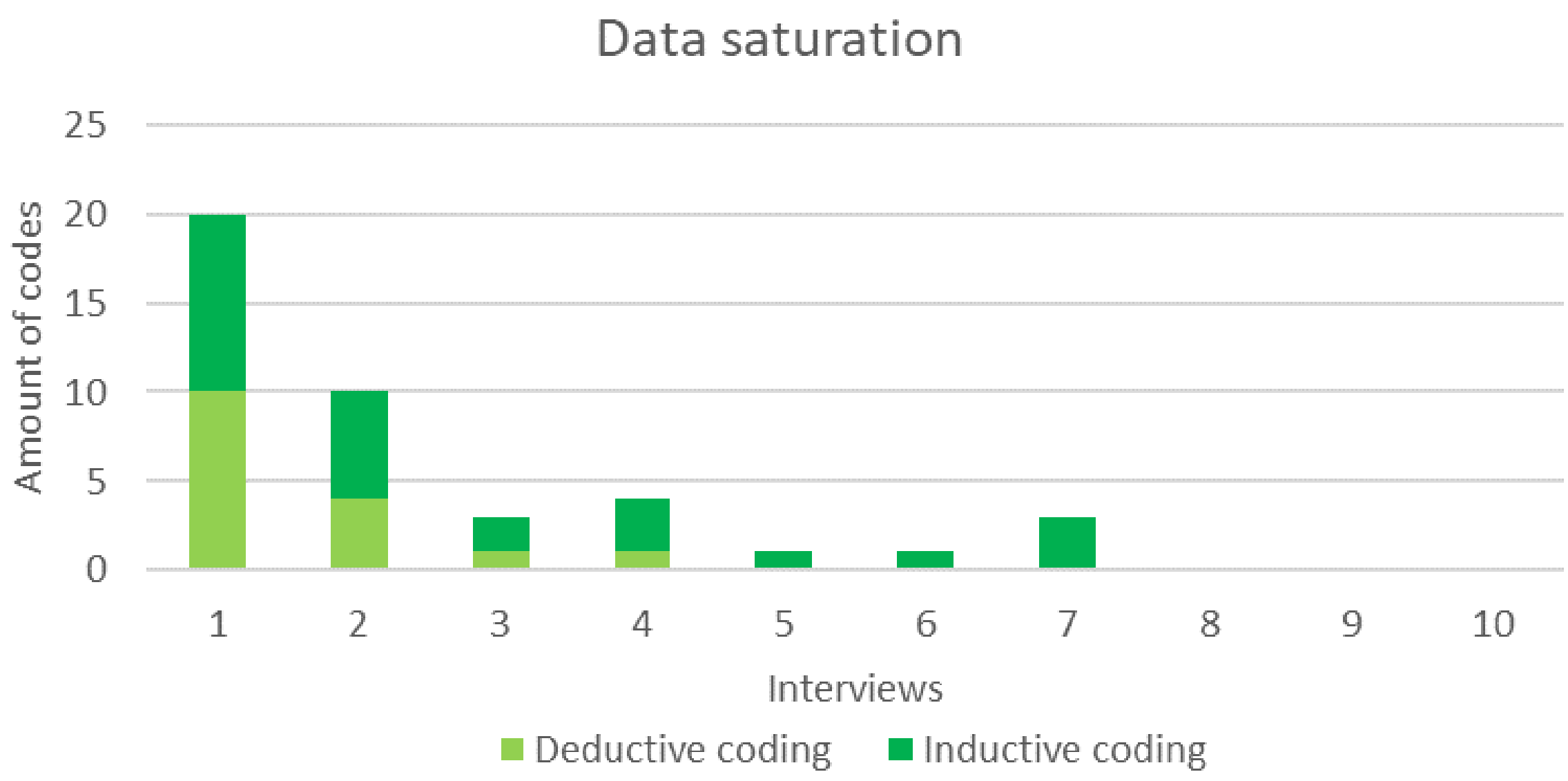

Supplement: Supplementary file 6 [file 11096_2026_2159_MOESM6_ESM.pdf]
